# Right focal atrial tachycardia following closure of patent foramen ovale with an amplatzer septal occluder device: case report

**DOI:** 10.1093/ehjcr/ytaf507

**Published:** 2025-10-08

**Authors:** Ahmed Makni, Jerome Bouet, Sophia Mzoughi, Jerome Taieb

**Affiliations:** Cardiology Department, Aix-en-Provence Hospital Center, Avenue des Tamaris, Aix en Provence 13100, France; Cardiology Department, Aix-en-Provence Hospital Center, Avenue des Tamaris, Aix en Provence 13100, France; Cardiology Department, Aix-en-Provence Hospital Center, Avenue des Tamaris, Aix en Provence 13100, France; Cardiology Department, Aix-en-Provence Hospital Center, Avenue des Tamaris, Aix en Provence 13100, France

**Keywords:** Right focal atrial tachycardia, Amplatzer septal occlude, Ablation, 3D mapping, Case report

## Abstract

**Background:**

Transcatheter closure of patent foramen ovale (PFO) is increasingly performed after cryptogenic stroke, but it may predispose to atrial arrhythmias. We report a rare case of late-onset right focal atrial tachycardia following PFO closure with an Amplatzer septal occluder device.

**Case summary:**

A 76-year-old man with no prior arrhythmia history underwent percutaneous PFO closure with a 25 mm Amplatzer device after a cryptogenic stroke. Three years later, he presented with symptomatic palpitations. Electrocardiography showed a focal atrial tachycardia. Electrophysiologic study and 3D mapping revealed a right atrial tachycardia originating at the site of the Amplatzer device scar. Radiofrequency ablation at this site successfully terminated the tachycardia, with no recurrence during follow-up.

**Discussion:**

This case illustrates the potential for late-onset right atrial tachycardia due to scarring and altered conduction around the PFO occluder. The site-specific reentry mechanism and delayed presentation may be related to progressive endothelialization of the device. To our knowledge, this is the first reported case of right atrial tachycardia occurring years after PFO closure and successfully treated with ablation.

**Take Home Messages:**

Radiofrequency ablation is a safe and effective treatment for device-related arrhythmias, offering symptom resolution and potentially preventing recurrence.

Learning points

- Right atrial tachycardia can occur late after PFO closure and may originate from scar tissue at the edge of the Amplatzer septal occluder.
- Electroanatomic mapping is essential to identify the arrhythmogenic substrate, which in this case was located on the right side of the device.
- Catheter ablation targeting the peridevice scar can be an effective and curative treatment for such device-related arrhythmias.

## Introduction

An increasing number of patients have undergone transcatheter device closure of patent foramen ovale (PFO). We report the case of a 76-year-old man who developed right focal atrial tachycardia following PFO closure with an Amplatzer septal occluder device.

## Summary figure

**Figure:**
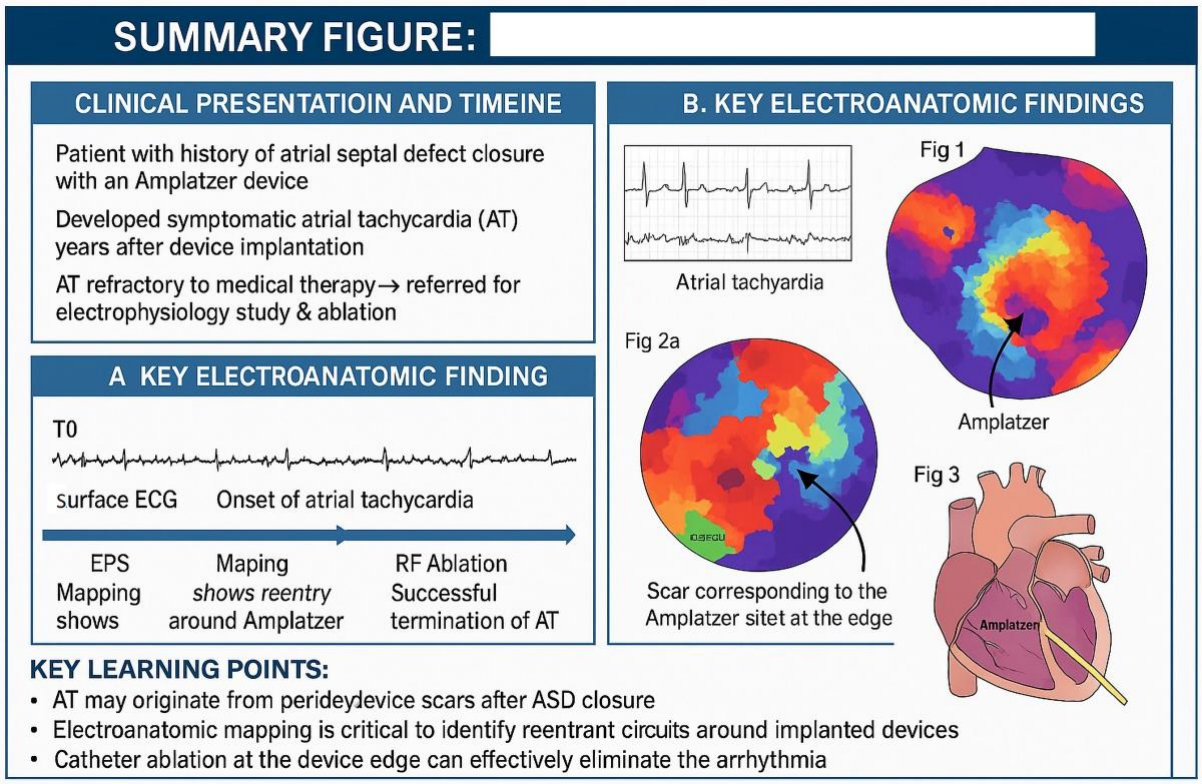


## Case presentation

A 76-year-old man underwent a catheter closure of the PFO with a 25-millimetre Amplatzer septal occluder device after a cryptogenic stroke. The indication for closure was a cryptogenic ischaemic stroke with no other identifiable cause after thorough evaluation. Echocardiography revealed a large right-to-left shunt at rest associated with an atrial septal aneurysm, considered high-risk features for paradoxical embolism. After multidisciplinary discussion, percutaneous PFO closure was proposed to prevent recurrence. There was no history of arrhythmias before the device closure.

**Figure 1:**
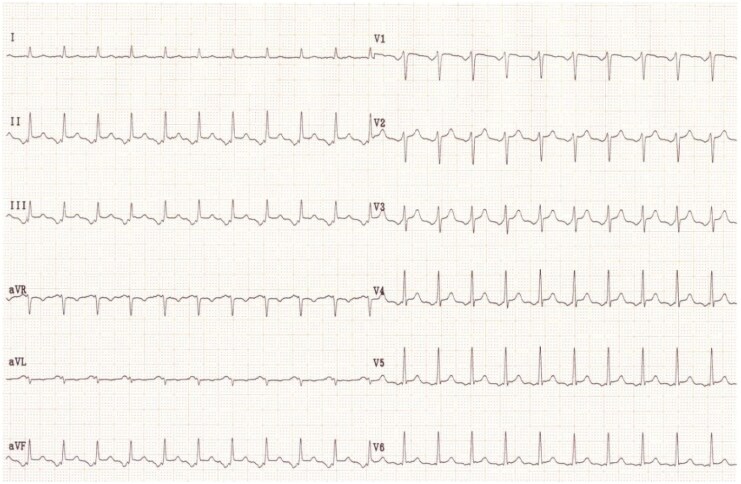
Surface ECG showing atrial tachycardia.

**Figure 2:**
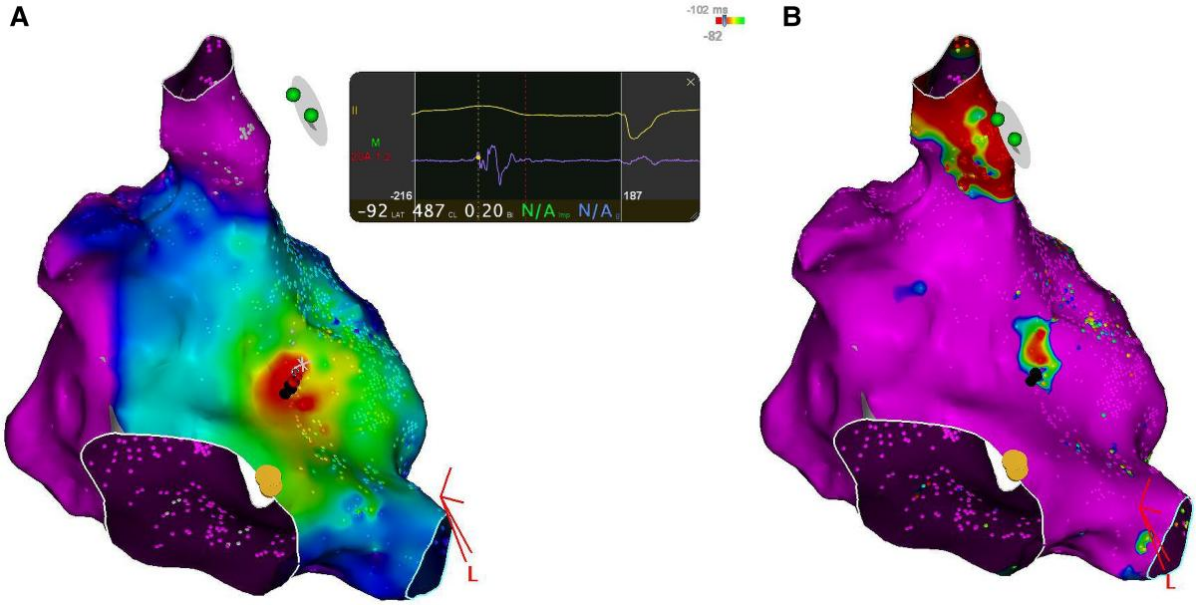
(*A*) Activation map demonstrating early depolarization on the right side of the Amplatzer device. (*B*) Bipolar voltage map showing scar corresponding to the Amplatzer site, with low voltage zones and earliest activation at the edge.

**Figure 3:**
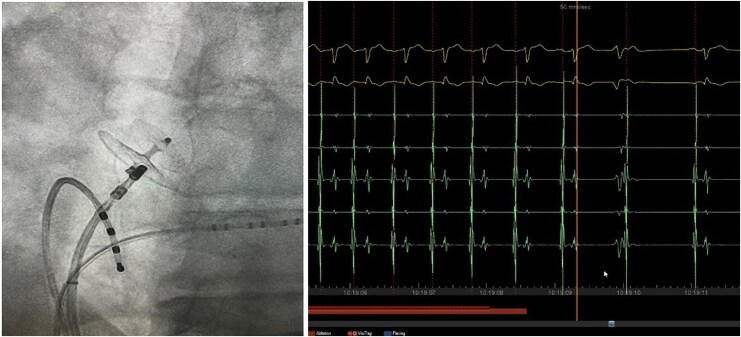
Catheter ablation at the edge of the Amplatzer device successfully terminating atrial tachycardia.

Three years later, he presented with symptomatic palpitations, and an electrocardiogram indicated focal atrial tachycardia (*Figure 1*). Electrophysiologic maneuvers were performed to differentiate the mechanism of supraventricular tachycardia and to exclude alternative diagnoses, including atrioventricular reentrant tachycardia. 3 D Mapping revealed a right atrial tachycardia at the level of the Amplatzer prosthesis (*Figure 2*) Three-dimensional electroanatomic mapping was performed using the CARTO® 3 system (Biosense Webster, Diamond Bar, CA, USA). At the successful ablation site, the unipolar electrogram showed a QS morphology, consistent with a focal activation pattern. Coronary sinus activation progressing from proximal to distal. Radiofrequency ablation of this focal right atrial tachycardia was successfully performed the earliest activation was recorded 28 ms prior to the onset of the *P*-wave on surface ECG. Entrainment maneuvers excluded macro-reentry, and the activation map showed a centrifugal pattern consistent with a focal origin. The mechanism was therefore confirmed as focal tachycardia in the electrophysiology laboratory. (*Figure 3*). No recurrence was observed during follow-up

## Discussion

PFO closure has been linked to a higher incidence of atrial arrythmia: 6% symptomatic AF in Close study,^1^ and 20% asymptomatic supraventricular tachycardia.^2^

Arrhythmias may be induced by these devices due to atrial scarring and sluggish conduction.^3^

Ablation of Atrial fibrillation on left atrium through Amplatzer has been described.^2^

Zaho reported a case of septal left atrial flutter after implantation of atrial septal occluder.^4^

In this case, the atrial tachycardia emerges from the right septum on the precise site of amplatzer scar which appears on activation map and voltage map (*Figure 2A* and *B*).

We hypothesize that the progressive endothelialisation of the septal occluder device could explain the site of microreentry and the 3 years delay of occurrence.

Interestingly a single application could stop the tachycardia which confirm that right septum scar and cicatrization is the exclusive mechanism

To the best of our knowledge, this is the first report of late-onset right atrial tachycardia occurring after PFO closure.

## Conclusion

This case highlights the possibility of right atrial tachycardia secondary to device closure of PFO and successful ablation.

## Lead author biography


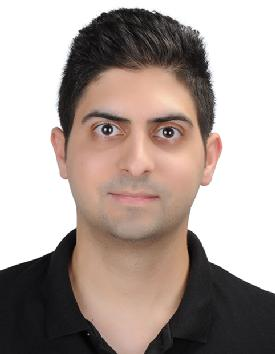


Dr. Ahmed Makni is a cardiologist affiliated with the Centre Hospitalier du Pays d'Aix in Aix-en-Provence, France.

**Consent:** Written informed consent was obtained from the patient for publication of this case report and any accompanying images. This report was prepared in compliance with the Committee on Publication Ethics guidelines.

**Funding:** This article did not receive any specific grant from funding agencies in the public, commercial, or not-for-profit sectors.

## Data Availability

The data underlying this article are available in the article itself. No additional data are available.
